# Improved water supply infrastructure to reduce acute diarrhoeal diseases and cholera in Uvira, Democratic Republic of the Congo: Results and lessons learned from a pragmatic trial

**DOI:** 10.1371/journal.pntd.0012265

**Published:** 2024-07-03

**Authors:** Karin Gallandat, Amy Macdougall, Aurélie Jeandron, Jaime Mufitini Saidi, Baron Bashige Rumedeka, Espoir Bwenge Malembaka, Andrew S. Azman, Didier Bompangue, Simon Cousens, Elizabeth Allen, Oliver Cumming

**Affiliations:** 1 Department of Disease Control, London School of Hygiene and Tropical Medicine, London, United Kingdom; 2 Department of Medical Statistics, London School of Hygiene and Tropical Medicine, London, United Kingdom; 3 Ministère de la Santé Publique, Division Provinciale de la Santé du Sud-Kivu, Zone de Santé d’Uvira, Uvira, Democratic Republic of Congo; 4 Department of Epidemiology, Johns Hopkins University, Baltimore, Maryland, United States of America; 5 Centre for Tropical Diseases and Global Health (CTDGH), Université Catholique de Bukavu, Bukavu, Democratic Republic of the Congo; 6 Geneva Centre for Emerging Viral Diseases, Geneva University Hospitals, Geneva, Switzerland; 7 Division of Tropical and Humanitarian Medicine, Geneva University Hospitals, Geneva, Switzerland; 8 Service d’Ecologie et Contrôle des Maladies Infectieuses, Faculté de Médecine, Université de Kinshasa, Kinshasa, Democratic Republic of Congo; 9 Department of Infectious Disease Epidemiology, London School of Hygiene and Tropical Medicine, London, United Kingdom; Natural History Museum, UNITED KINGDOM OF GREAT BRITAIN AND NORTHERN IRELAND

## Abstract

**Background:**

Safely managed drinking water is critical to prevent diarrhoeal diseases, including cholera, but evidence on the effectiveness of piped water supply in reducing these diseases in low-income and complex emergency settings remains scarce.

**Methods:**

We conducted a trial of water supply infrastructure improvements in Uvira (DRC). Our primary objective was to estimate the relationship between a composite index of water service quality and the monthly number of suspected cholera cases admitted to treatment facilities and, as a secondary analysis, the number of cases confirmed by rapid diagnostic tests. Other exposures included the quantity of supplied water and service continuity. We used Poisson generalised linear models with generalised estimating equations to estimate incidence rate ratios.

**Findings:**

Associations between suspected cholera incidence and water service quality (RR 0·86, 95% CI 0·73–1·01), quantity (RR 0·80, 95% CI 0·62–1·02) and continuity (RR 0·81, 95% CI 0·77–0·86) were estimated. The magnitudes of the associations were similar between confirmed cholera incidence and water service quality (RR 0·84, 95% CI 0·73–0·97), quantity (RR 0·76, 95% CI 0·61–0·94) and continuity (RR 0·75, 95% CI 0·69–0·81). These results suggest that an additional 5 L/user/day or 1.2 hour per day of water production could reduce confirmed cholera by 24% (95% CI 6–39%) and 25% (95% CI 19–31%), respectively.

**Interpretation:**

Ensuring a sufficient and continuous piped water supply may substantially reduce the burden of endemic cholera and diarrhoeal diseases but evaluating this rigorously is challenging. Pragmatic strategies are needed for public health research on complex interventions in protracted emergency settings.

**Trial registration:**

The trial is registered in ClinicalTrials.gov ID NCT02928341. https://classic.clinicaltrials.gov/ct2/show/NCT02928341.

## Author summary

Diarrhoeal diseases including cholera remain a leading cause of mortality and morbidity, especially in areas with limited access to safe water, sanitation and hygiene (WASH). However, evidence for the effect of piped water supply in reducing diarrhoeal diseases and cholera in complex emergency settings remains scarce. We evaluated a large-scale infrastructure programme to improve and expand the piped drinking water supply service in the town of Uvira (South Kivu, Democratic Republic of the Congo), an area with a high burden of cholera and diarrhoeal diseases. We used five years of data from clinical surveillance and systematic cholera confirmation by rapid diagnostic tests in order to estimate associations between water supply infrastructure interventions and the incidence of diarrhoeal diseases and cholera. Our findings are consistent with existing evidence that a high-quality (or “safely managed”) water service can be effective in reducing the burden of diarrhoeal diseases and cholera in low-income urban settings. Beyond overall service quality, the quantity of water supplied to the household and the continuity of the drinking water supply were both associated with a reduced risk of cholera.

## Introduction

Diarrhoeal diseases remain a leading cause of mortality and morbidity worldwide, with aetiology varying by age and setting [1]. Cholera is an acute diarrhoeal disease caused by toxigenic *Vibrio cholerae* O1 or O139. Modelling suggests that 2·9 million cholera cases (95% CI: 1·3–4·0) and 95 000 deaths (95% CI: 21,000–143,000) occur annually, primarily in Africa and Asia [2]. In sub-Saharan Africa, over half of the cases are concentrated in high-burden “hotspots”, which represent less than 4% of the population in the region [3]. Frequent underreporting and a low-specificity standard case definition, combined with limited surveillance capacity, make it challenging to accurately estimate the respective burdens of cholera and other acute diarrhoeal diseases [2,4].

The Global Task Force on Cholera Control has developed a roadmap to achieve a 90% reduction in cholera mortality by 2030 [5]. The multisectoral cholera elimination strategy prioritises targeted interventions in cholera hotspots along three pillars: a) surveillance and health systems strengthening; b) oral cholera vaccination (OCV); and, c) long-term improvements in water, sanitation and hygiene (WASH) access. The third component aligns closely with the United Nations’ Sustainable Development Goal (SDG) indicators 6.1 and 6.2 to achieve universal access to safely managed water and sanitation services by 2030 [6].

Globally, in 2019, 69% of the diarrhoeal disease morbidity burden and 1·04 (95%CI: 0·93–1·16) million deaths due to diarrhoeal diseases were estimated to be attributable to inadequate WASH access [7]. In 2020, 771 million people lacked access to basic drinking water services, including almost half in Sub-Saharan African countries with a high burden of diarrhoeal diseases [8]. Improving access to safely managed water services can prevent waterborne transmission of diarrhoeal diseases through microbiologically safe drinking water and increased water availability for personal and domestic hygiene. The latter may be critical for cholera control given the importance of within-household transmission during outbreaks [9,10].

Although historical evidence suggests that water and sanitation infrastructure contributed to mortality reductions from infectious diseases in Europe and North America, there is limited scientific literature on the impacts of water supply infrastructure improvements intended to reduce the burden of diarrhoeal diseases and cholera [11,12]. The health impacts of WASH interventions in complex emergency settings also remain under-researched [13]. Besides accessibility challenges, these gaps may be due to the methodological, logistical and financial challenges related to the study of complex interventions, which are exacerbated in the resource-limited and often unstable contexts where the cholera burden is concentrated.

In 2014, a major infrastructure programme was initiated to improve the piped water supply in Uvira, a town located within a cholera hotspot in Eastern Democratic Republic of the Congo (DRC), in line with the national multisectoral cholera elimination plan 2013–2017 [3,14]. The programme was led by REGIDESO, the national water utility, with support from the Agence Française de Développement (AFD), Veolia Foundation (VF), and the European Union. We investigated the relationship between changes in the piped water supply service and the incidence of suspected and confirmed cholera cases admitted to the main cholera treatment facilities of the town.

## Methods

### Ethical approval

The trial was registered on clinicaltrials.gov (NCT02928341, 10th October 2016, https://clinicaltrials.gov/ct2/show/NCT02928341) and received ethics approval from the London School of Hygiene and Tropical Medicine (8913, 10603 –and amendments) and the Ethics Committee of the School of Public Health, University of Kinshasa, Democratic Republic of the Congo (ESP/CE/088/2015, renewed annually, and amendments). Written consent was obtained from all participants prior to sample collection and written parental consent was sought for patients under 15 years old.

### Study design

We designed a pragmatic stepped-wedge, cluster randomised trial (SW-CRT) to assess the impact of water supply infrastructure improvements on acute diarrhoeal diseases and cholera in Uvira, with concurrent process and economic evaluations [15]. This manuscript focuses on the epidemiological analysis of trial data; the process evaluation, including household survey results on domestic WASH practices, and the cost-of-illness study are described in detail elsewhere [16,17].

In the previously published trial protocol, and in light of the complex setting, we pre-specified different options for analysis dependent on whether specific identified risks to implementation manifested themselves [15]. As described below, several of these risks materialised and the implementation of the intervention diverged from the randomised allocation of the stepped-wedge trial design. Consequently, analysing the data according to the planned allocation would not have been appropriate and we selected an alternative approach in line with the options outlined in the protocol, treating this as an observational study.

### Study setting

Uvira is a town of approximately 280,000 inhabitants (according to 2020 official records) and a cholera-endemic areas in Eastern DRC [18]. It is affected by protracted conflict and continuing population displacements [19,20]. The Uvira water supply network was built during the colonial era and severely damaged during the First Congo War (1996–1997) [21]. Before the start of the water supply infrastructure improvement programme in 2018, approximately 30% of Uvira’s population had access to an intermittent piped water service with frequent disruptions [21]. In surveys conducted by the study team in 2016 and 2017, almost half (47%) of a representative sample of residents (747 respondents from 458 households) reported using surface water, about a third (34%) used a tap outside their compound, and 18% had a tap in their compound as their primary drinking water source [22].

In April 2020, extreme flooding events in the town of Uvira affected an estimated 86,000 people and destroyed the main intake for the piped water supply network [23,24]. This led to a 6-week interruption in the water supply service and a sustained reduction in the production capacity of the water treatment plant–until the installation of a new water intake, commissioned in January 2024. A survey of 148 households suggested that a minority of the population (9%) were still using emergency water supply three months post-flooding, in July 2020 [16,25].

Towards the end of the infrastructure improvement programme, in a survey of 528 households conducted in September 2021, REGIDESO taps, either outside (42.6%) or in the compound (18.6%), and rivers (30.9%) were most commonly reported as main water sources [16,26], suggesting a slight increase (+9.2 percentage points) in the proportion of households using REGIDESO taps compared to the situation in 2016/17.

The main care structures for acute diarrhoeal diseases in Uvira are the cholera treatment centre (CTC) of the Hôpital Général de Référence d’Uvira located near the town centre and the cholera treatment unit (CTU) of the Kalundu CEPAC health centre, the latter having opened in July 2019 approximately 6 km south of the CTC. Both provide free treatment for patients of any age, including rehydration and, in some cases, antibiotics [27].

A mass OCV campaign was deployed in Uvira in two rounds, from 29^th^ July to 5^th^ August and from 1^st^ to 8^th^ October 2020. The licensed oral vaccine Euvichol-Plus was offered to all individuals aged one year or older. The administrative coverage reported by the Ministry of Health was 96.5% for at least one vaccine dose. Preliminary coverage estimates from a representative household survey conducted one year after vaccination ranged from 38.3 to 79.9% per cluster (Table A in S1 Table).

### Participants

The population of interest for the study was the residents of the town of Uvira, including all age groups, as the water supply improvement programme was intended to benefit all Uvira inhabitants. Population numbers per *avenue* (the smallest administrative unit) were available for November 2017 from Uvira authorities [22]; the population distribution between avenues was assumed to remain constant over time and combined with more recent official records in order to estimate the population size by cluster in subsequent years (Table 1).

**Table 1:** Estimatead population size, suspected and confirmed cholera cases by cluster.

	Suspected cholera cases		Confirmed cholera cases		2021 population size (%)	
n	4556		1503		286931	
Age (median [IQR])	20.0 [9.0, 40.0]		21.0 [10.0–40.0]			
Sex = M (%)	2277 (50.0)		779 (51.8)			
**Cluster (%)**						
1	66	(1.4)	14	(0.9)	4688	(1.6)
2	168	(3.7)	50	(3.3)	5452	(1.9)
3	250	(5.5)	75	(5.0)	12212	(4.3)
4	252	(5.5)	97	(6.5)	13387	(4.7)
5	251	(5.5)	92	(6.1)	15462	(5.4)
6	224	(4.9)	79	(5.3)	9044	(3.2)
7	51	(1.1)	20	(1.3)	6453	(2.2)
8	39	(0.9)	13	(0.9)	12086	(4.2)
9	520	(11.4)	180	(12.0)	11326	(3.9)
10	203	(4.5)	73	(4.9)	12787	(4.5)
11	551	(12.1)	197	(13.1)	53528	(18.7)
12	184	(4.0)	59	(3.9)	14975	(5.2)
13	476	(10.4)	148	(9.8)	52509	(18.3)
14	259	(5.7)	74	(4.9)	19376	(6.8)
15	337	(7.4)	119	(7.9)	22257	(7.8)
16	725	(15.9)	213	(14.2)	21389	(7.5)

In order to estimate suspected and confirmed cholera incidence in the town of Uvira, anonymised patient information as recorded in the CTC/CTU registry, including age, sex, address, dates and length of stay were shared with the study team by Uvira health authorities. All patients admitted to the CTC and CTU with acute watery diarrhoea (i.e. passing 3 or more loose or watery stools in 24 hours) were considered suspected cholera cases and eligible for participation in the study. From April 2016, participants were recruited continuously to collect a rectal swab for enrichment in alkaline peptone water (Himedia, India) for 6 hours followed by cholera confirmation using rapid diagnostic tests (RDT) (Crystal VC O1/O139, Arkray Inc., India). Rectal swabs were selected to minimise the risk of contamination and the enrichment procedure was estimated in a previous study to have a sensitivity of 92% and a specificity of 91% [27,28]. A recent systematic review found pooled estimates of 83% (67–92%) and 98% (94–99%) for sensitivity and specificity, respectively, for testing of APW-enriched samples using VC Crystal kits [29]. Enrolment was on a continuous basis, 7 days a week, with testing performed during daytime, typically within 24 hours or less following admission. No restriction was placed on patient age. Written consent was obtained from all participants prior to sample collection and written parental consent was sought for patients under 15 years old.

### Intervention

The intervention was an infrastructure programme to expand and improve the water supply system in Uvira. This system included a water intake on the Mulongwe river feeding into a water treatment plant. Treated water was pumped into a tank located above the town, which then supplied water to users through gravity flow. The intervention and resulting changes in water service quality have been described in detail elsewhere [30]. In summary, it included renovating and doubling the capacity of water production facilities; building a new water tank (2 000 m^3^) to supplement the existing one (1 600 m^3^); and, rehabilitating and installing new pipes (approx. 34 km in total). In addition, between September 2019 and December 2021, 1 191 households were newly connected to the water network, 717 private taps were rehabilitated, and 56 community taps were built; the installation of new taps was organised by cluster and represented the most visible part of the intervention. All changes to the water supply network including date of completion and location (GPS coordinates) were documented by the construction teams in each cluster. Additionally, operational and billing information was shared by REGIDESO, including monthly volumes of water produced and billed to domestic users.

### Randomisation and masking

The town of Uvira was divided into 16 clusters of varying areas (0.36–2.75 km^2^) and population sizes (3,996–56,106 in 2018), based on the length of pipes to be installed or rehabilitated, the number of new community taps, as well as alignment with administrative and natural boundaries (e.g. rivers). Clusters were randomised to different initiation periods for the installation of household and community taps [15]. The address (combination of avenue, neighbourhood and health area) of patients admitted to the CTC/CTU was used to determine which cluster they came from.

Clusters were allocated by the research team using a random number generator. The intervention interval–or “step”–during which construction works for the installation of new private and community taps were to be implemented in each cluster was up to eight weeks. However, multiple challenges (supply chain disruptions and border closures related to the Covid-19 pandemic, a rise in the level of Lake Tanganyika, extreme flooding events in April 2020) caused substantial delays and required changes to the order of intervention. As a result, the randomised cluster allocation could not be followed and the duration of works in each cluster exceeded eight weeks in all but two clusters.

Blinding of participants was not possible due to the nature of the intervention: new piped water connections were installed on demand, and construction works are visible.

### Exposure

According to SDG 6.1 and World Health Organisation (WHO) definitions, a water supply service should be accessible on premises, available when needed, affordable, and provide a sufficient quantity of safe water to cover drinking, personal and domestic hygiene needs [6,31]. In order to understand how the programme in Uvira impacted piped water supply over time, an index was developed based on a fuzzy inference method to describe the monthly water service quality in each cluster on a scale of 0–100%, where 100% corresponds to a ‘safely managed’ water supply service in line with the SDG 6.1 definition. Four components were considered [30]: (i) accessibility, defined as the proportion of houses that have a private tap or are located within 200 m of a community tap, based on GPS coordinates for existing and new taps shared by the construction team, and buildings extracted from high-resolution satellite imagery; (ii) water quantity, estimated based on the average volume billed per user, according to REGIDESO billing records; (iii) service continuity, approximated by the daily hours of pumps operation at the water treatment and pumping station, based on REGIDESO operational records; and, (iv) affordability, defined as the proportion of users paying less than 5% of the average monthly household income for their water service, based on REGIDESO billing records. Quantitative inputs for each component were transformed into categorical inputs (very low, low, medium, high or very high). A tailored inference system of 25 rules was then used to determine a categorical value for the index, which was transformed into a percentage value through the centre of gravity defuzzification method. The rules were defined in such a way that accessibility and quantity take precedence over continuity and affordability, because the last two dimensions are only meaningful to consider if the water supply service is first accessible and available. The index is therefore strongly influenced by the “water quantity” component in the Uvira context, while simulations for a broad range of conditions confirmed that it would adequately reflect changes in each of the four components [30]. This index is the main exposure of interest, because it summarises in a single value multiple aspects of the water supply service relevant to our study. The individual index components were considered as secondary exposures.

### Outcomes

The primary study outcome was the monthly incidence of suspected cholera cases, measured by the number of CTC/CTU admissions attributed to each cluster, which includes patients affected by a range of acute diarrhoeal diseases [32]. The monthly incidence of confirmed cholera cases, based on RDT results for eligible, consenting patients admitted as suspected cholera cases to the CTC/CTU was a secondary outcome.

### Statistical analyses

In our primary analyses the dependent variable was monthly incidence of suspected cholera (primary outcome), and the monthly water supply service index was the independent variable. In secondary analyses the independent variable was each of the components of this composite measure: accessibility, water quantity, service continuity, and affordability. Analyses were also carried out in which the dependent variable was confirmed cases of cholera only (secondary outcome).

We used Poisson generalised linear models with an offset, for the annual population size per cluster, to estimate rate ratios. Models produced estimated incidence rate ratios corresponding to a five unit increase in each of the measures of water supply. The magnitude of the effects was contingent on the choice of a five unit increase in exposure as reference, which was considered reasonable based on the scales of the measures. Inference was performed using Generalized Estimating Equations (GEE) with robust standard errors to account for within cluster correlations over time. The offset for the annual population size per cluster and robust GEE model mitigate the influence of varying population densities across clusters. Given the small number of clusters (n = 16), a small sample correction was also applied [33].

To account for the seasonal pattern of cholera rates over the course of a year, a harmonic form for time in months was included.

Linear and quadratic terms for year were also included to account for trends over time. Suspected and confirmed cholera rates were observed to vary between years, so an interaction between the linear term for year and the harmonic terms for time in months was included. The following additional covariates were included in all models: median distance to cholera treatment facilities from each cluster (in km), an estimated percent coverage of the oral cholera vaccine at the cluster level based on preliminary results from a representative survey conducted in August 2021 [34] and, when the outcome was confirmed cholera incidence, the monthly proportion of patients tested in each cluster. Statistical variable selection was not carried out. All covariates were selected based on expert opinion.

The analyses were carried out using data from January 2017 to December 2021, which is the period for which detailed information on the water supply service was available. A sensitivity analysis was carried out to assess the impact of modelling time in years with a quadratic term compared to using a categorical term: all covariates were added in the same way as described above, except ‘year’, for which a categorical term was included. The R statistical software environment was used with packages ‘geepack’ and ‘saws’ [33,35,36].

## Results

A total of 4556 suspected cholera cases were admitted to cholera treatment facilities in Uvira and enrolled in the study between January 2017 and December 2021 (Table 1). The majority (92·3%) sought care at the General Hospital CTC and 50.0% were recorded as female. Age was available for 4463 (97·9%) patients; the median was 20.0 years (IQR 9.0–40.0) and patients aged five years or younger represented 16·4% of admitted cases (compared to an estimated 12.8% of the Uvira population based on 2019 official records). Twenty-three deaths were recorded among patients admitted to cholera treatment facilities between 2017 and 2021, including seven in patients who tested positive for cholera.

**Fig 1:**
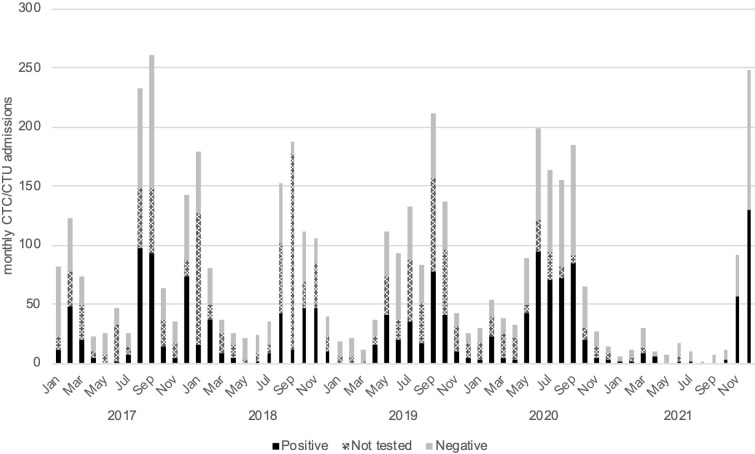
Monthly incidence of suspected and confirmed cholera in Uvira, DRC by rapid diagnostic test outcome (colour).

Rectal swabs were collected from 3309 patients (72·6%). The proportion of patients tested varied between 63% in 2018 and 89% in 2021 (Table B in S1 Table). The main reason for missing tests was the absence of the responsible laboratory technician. Among samples subjected to rapid diagnostic tests, 45·4% were positive (Fig 1). The average monthly incidence rate of suspected cholera across the entire town of Uvira was 4·0 per 10 000 inhabitants. When conservatively categorising untested suspected cases as rapid test negative, the mean monthly incidence rate of confirmed cholera was estimated to be 1·3 per 10 000. Variations in incidence rates were observed between clusters and over time (Table C in S1 Table and Fig A in S1 Fig).

The water supply service quality as described by an index from 0 to 100%–the main exposure of interest in our analysis–ranged from 6·7% (“very low”) to 38·1% (“medium-low”) by cluster in January 2017, prior to the start of construction works in Uvira. The observed change between January 2017 and December 2021 ranged from +1·8% to -18.3%. The change was negative or null in 14 out of 16 clusters, with an average of -5·4%, a median of -3·0%. In December 2021, the index ranged from 6·7% (very low) to 25·0% (low). This evolution was primarily driven by the limited quantity of water available and increasing frequency of water supply interruptions. The estimated volume of water delivered remained under 20 L per capita per day during >95% of the observation period and showed a decreasing trend, as the number of connections to the network substantially increased (with the “accessibility” component of the index reaching 100% in four clusters) while production capacity remained limited (Fig B in S1 Fig). Pumps operated 58% of the time on average and electricity supply deteriorated over time, leading to reduced hours of operation and more frequent service interruptions [16].

**Fig 2:**
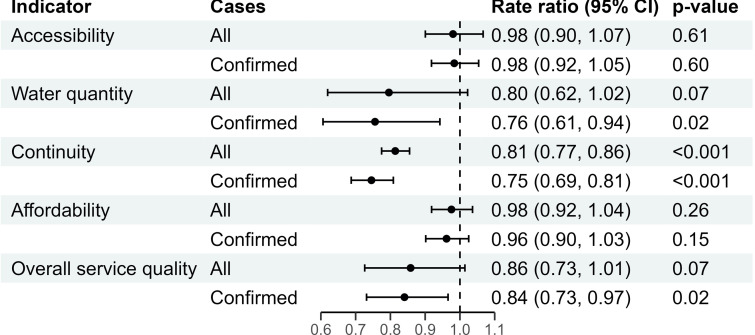
Rate ratios for 5-unit increases in accessibility, continuity, affordability, and overall service quality, or a 5 L/p/day increase in water quantity for both all suspected cholera cases and those who were positive with a rapid diagnostic test (“confirmed”).

Overall service quality was associated with confirmed cholera incidence (RR 0·84, 95% CI 0·73–0·97, p = 0·024), suggesting that a 5-percentage point increase in the water service quality index could reduce confirmed cholera incidence by 16%. Concretely, in Uvira, such an increase in the index value could in most cases be achieved by increasing the quantity of water available to users. Water quantity (RR 0·76, 95% CI 0·61–0·94, p = 0·020) and service continuity (RR 0·75, 95% CI 0·69–0·81, p<0.001) were also individually associated with confirmed cholera (Fig 2), meaning that increasing the quantity of water available by 5 L per user per day or increasing monthly pumping hours by 5% (i.e. approximately 1.2 hour per day) were associated with a 24% and 25% reduction in the incidence of confirmed cholera, respectively.

Rate ratio estimates were similar for suspected cholera incidence and the strongest association was with service continuity, a 5% increase in pumping hours associated with a 19% rate reduction (RR 0·81, 95% CI 0·77–0·86, p<0.001) (Fig 2).

Neither confirmed nor suspected cholera incidence was associated with accessibility or affordability of the water service (Fig 2).

A sensitivity analysis to assess the impact of modelling secular trend using a categorical form, compared to the quadratic form used in the main model, suggested that the only association that would substantially change and become weaker is that between continuity of supply and suspected cholera cases (Fig C in S1 Fig).

## Discussion

We studied a large-scale intervention to improve piped water supply in Uvira (DRC). Our results support the case that water supply service improvements can reduce acute diarrhoeal diseases and, in particular, cholera. They provide evidence based on five years of water supply monitoring and clinical surveillance in a town with endemic cholera and a high burden of diarrhoeal diseases. Challenges in Uvira are typical of those found across cholera hotspots in the broader region, including vulnerability to climate-related natural hazards, protracted conflict, constant population movements, and multiple health emergencies [37].

The comparison of different water supply service components underlines the importance of water quantity and service continuity. The association with continuity, although sensitive to assumptions about seasonality, is consistent with previous analyses of data from the same setting [38]. It is a reminder that the quality of water supply services, beyond infrastructure improvements, is critical to reduce the disease burden, and suggests that an incremental approach to improving water supply services is unlikely to yield significant progress in reducing cholera and other acute diarrhoeal diseases in settings such as Uvira. Our results thus provide support for the higher level of service envisaged under SDG 6. Concretely, in order to achieve public health and other benefits, programmes aiming to prevent diarrhoeal disease and cholera in urban settings through an improved piped water supply should: (i) design and invest in infrastructure solutions that can deliver a “safely managed” water service in the long term, resilient to a changing climate; and (ii) integrate sufficient technical and organisational support to water utilities to ensure an adequate system operation. In the Uvira context, where water service intermittence is closely linked to electricity supply issues, our results also call for enhanced cross-sectoral planning and coordination [16].

Our results were similar for confirmed and for suspected cholera (i.e. acute diarrhoeal diseases), with slightly smaller effects for the latter that could reflect the variable susceptibility of different pathogens to water supply service improvements. Case confirmation via rapid diagnostic tests suggests that a substantial proportion of the patients admitted to cholera treatment facilities in Uvira are not infected by *Vibrio cholerae* O1 [27]. A wide range of enteric pathogens circulate in Uvira, with enterotoxigenic *Escherichia coli*, *Cryptosporidium* spp. (resistant to chlorination), and *Campylobacter* (predominantly foodborne) detected most frequently among patients admitted to the CTC after *V*. *cholerae* [32]. This diversity is typical of settings with a high burden of diarrhoeal disease [1].

There are three main limitations to our study related to (i) implementation challenges, (ii) documentation of the exposure of interest, and (iii) care-seeking behaviour.

We had initially planned to conduct a SW-CRT whereby new taps would be installed in a randomised order across the town of Uvira [15]. However, due to multiple disruptions (extreme flooding, lake level rise, Covid-19), adherence to the intervention protocol was not possible. The selection of a SW-CRT design was based on the considerations that it would strengthen our ability to draw causal inferences and *a priori* be compatible with a sequential roll-out of the construction works. As a consequence of the implementation challenges and of using an observational approach for the analysis, we could neither clearly distinguish situations before and after the intervention nor evaluate the impact of the intervention. Instead, the focus was on observed variations in the water supply service (exposure) and estimated cholera and diarrhoeal disease incidence (outcomes), amidst complex influences. Population movement is an important factor which could not be documented systematically and integrated into our modelling. However, our experience suggests that realistic expectations should be set, and pragmatic approaches adopted, for the rigorous evaluation of non-clinical, complex interventions in challenging emergency settings. In particular, pre-specifying risks and analysis strategies for different scenarios as we did in the published protocol for this study [15] reduces potential bias.

A second limitation lies in the documentation of the exposure of interest, namely changes in the water supply service. The index used in our analyses is primarily based on utility data, which were collected retrospectively and may not always reflect users’ experiences. Continuity was approximated for the entire town by a centralised, indirect measure (hours of operation of the pumps). The continuous monitoring of water quality was not possible–water samples were only collected and tested during household surveys–and water quality is a critical dimension of the water supply service quality that is not included in our analyses.

A third limitation is the reliance of our study on clinical surveillance based at the official cholera treatment facilities, which overlooks the multiplicity of private and informal options available for diarrhoeal disease and cholera care in Uvira and may have been impacted by changes in care-seeking behaviour over time linked to the Covid-19 pandemic. Among respondents who reported seeking care for diarrhoea in a survey conducted in September 2021 in Uvira (n = 160), 70% said that they went to a pharmacy and less than 5% mentioned a public healthcare facility [16,26]. While it seems reasonable to assume that the most severe cases are referred to the main cholera treatment centre located at the Uvira General Hospital, three community deaths likely due to acute diarrhoea were reported in Uvira between September 2021 and January 2023 [39], and many cases certainly remained undetected in our study. While decreases in attendance of health services have been documented during the Covid-19 pandemic, including in Kinshasa [40], we were not able to assess such changes in our study. If possible, clinical surveillance should be complemented by community-based cholera detection in future research projects in order to provide a more reliable picture of the cholera and diarrhoeal disease burden.

We assessed the relationship between changes in the piped water supply service and diarrhoeal diseases in an urban setting with endemic cholera and a high diarrhoeal disease burden in the context of a largescale programme to improve water supply, in Eastern Democratic Republic of the Congo. Our results provide insights into the potential challenges and effects of water supply interventions in similar settings, where large investments in water supply and sanitation services will be necessary to accelerate progress towards SDG6 and the global target of cholera elimination by 2030. We found strong associations between the continuity of water supply and both confirmed and suspected cholera, as well as between the quantity of water delivered and confirmed cholera. This implies that water infrastructure programmes must achieve high standards of service–providing a sufficient, continuous water supply–in order to materialise expected public health benefits in terms of diarrhoeal disease prevention.

## Supporting information

S1 Foreign Language Abstract French translation of the abstract. (DOCX)

S1 CONSORT Checklist Supplementary materials 3: Checklist of information to include when reporting a stepped wedge cluster randomised trial (SW-CRT). (PDF)

S1 Table **Table A**: Estimated vaccination coverage, distance to closest CTC or CTU by cluster. **Table B:** Annual proportion of patients tested via rapid diagnostic tests among those admitted (% testing positive among those tested). **Table C:** Yearly incidence of suspected and confirmed cholera per 1,000 residents.
(DOCX)

S1 Fig **Fig A**. Monthly average number of suspected and confirmed cases from each cluster. **Fig B:** Description of water supply-related exposures: monthly mean accessibility (%), water quantity (L/cap/day), affordability (%), and overall service quality (composite index, %) by cluster and continuity (%) for the town. **Fig C:** Sensitivity analysis comparing two approaches to modelling time.
(DOCX)
