# Supplementary material for: Improved water supply infrastructure to reduce acute diarrhoeal diseases and cholera in Uvira, Democratic Republic of the Congo: Results and lessons learned from a pragmatic trial
Source: PLoS Negl Trop Dis. 2024 Jul 3;18(7):e0012265. doi: 10.1371/journal.pntd.0012265 (PMC11251581; doi:10.1371/journal.pntd.0012265)
Supplement: S1 Foreign Language Abstract — (DOCX) [file pntd.0012265.s001.docx]

***ABSTRACT – FRENCH TRANSLATION***

**RESUME**

**Contexte.**

Les services d'eau potable gérés en toute sécurité sont essentiels pour prévenir les maladies diarrhéiques, dont le choléra, mais les preuves scientifiques de l'efficacité des interventions d'approvisionnement en eau centralisé pour réduire ces maladies dans les contextes à faible revenu et d’urgences complexes restent limitées.

**Méthodes.**

Nous avons évalué des améliorations des infrastructures d’approvisionnement en eau à Uvira (RDC). Notre objectif principal était d’estimer la relation entre un index composite de la qualité du service d’approvisionnement en eau et le nombre mensuel de cas de choléra suspects admis dans les structures de soin ainsi que, en analyse secondaire, le nombre de cas de choléra confirmés par tests de diagnostic rapide. D’autres expositions incluaient la quantité d’eau fournie et la continuité du service. Nous avons utilisé un modèle linéaire généralisé de Poisson avec des équations d’estimation généralisées pour estimer les rapports de taux d’incidence.

**Résultats.**

Les associations entre l’incidence des cas de choléra suspects et le service d’approvisionnement en eau (RR 0·86, 95% CI 0·73-1·01), la quantité (RR 0·80, 95% CI 0·62-1·02) et la continuité (RR 0·81, 95% CI 0·77-0·86) ont été estimées. Les effets étaient similaires pour les associations entre les cas de choléra confirmés et le service d’approvisionnement en eau (RR 0·84, 95% CI 0·73-0·97), la quantité (RR 0·76, 95% CI 0·61-0·94) et la continuité (RR 0·75, 95% CI 0·69-0·81). Ces résultats suggèrent que fournir 5 L/p/j additionnels ou augmenter les heures de production de 1.2 heure par jour pourrait réduire l’incidence du choléra confirmé de 24% (IC 95%, 6-39%) et 25% (IC 95% CI 19-31%), respectivement.

**Interprétation.**

Assurer un approvisionnement en eau suffisant et continu peut réduire considérablement le fardeau du choléra et des maladies diarrhéiques endémiques mais cela reste difficile à évaluer rigoureusement. Des stratégies de recherche pragmatiques sont nécessaires pour entreprendre des études en santé publique sur des interventions complexes dans des contextes d’urgence prolongée.
