# Supplementary material for: Improved water supply infrastructure to reduce acute diarrhoeal diseases and cholera in Uvira, Democratic Republic of the Congo: Results and lessons learned from a pragmatic trial
Source: PLoS Negl Trop Dis. 2024 Jul 3;18(7):e0012265. doi: 10.1371/journal.pntd.0012265 (PMC11251581; doi:10.1371/journal.pntd.0012265)
Supplement: S1 Table — Table A: Estimated vaccination coverage, distance to closest CTC or CTU by cluster. Table B: Annual proportion of patients tested via rapid diagnostic tests among those admitted (% testing positive among those tested). Table C: Yearly incidence of suspected and confirmed cholera per 1,000 residents. (DOCX) [file pntd.0012265.s003.docx]

**SUPPLEMENTAL INFORMATION**

**S1 TABLES**

**Table A** Estimated vaccination coverage, distance to closest CTC or CTU by cluster.

| Cluster | OCV coverage for  at least 1 dose (%) after August 2020 | Distance to CTC  (until July 2019) | Distance to closest CTC/CTU (after July 2019) |
| --- | --- | --- | --- |
| 1 | 68.2 | 7884.0 | 7884.0 |
| 2 | 68.2 | 5967.0 | 5967.0 |
| 3 | 64.5 | 5107.2 | 5107.2 |
| 4 | 61.3 | 5120.7 | 5120.7 |
| 5 | 45.1 | 3651.5 | 3651.5 |
| 6 | 79.9 | 3909.7 | 3909.7 |
| 7 | 79.9 | 2637.8 | 2637.8 |
| 8 | 79.9 | 1988.1 | 1988.1 |
| 9 | 68.2 | 2095.9 | 2095.9 |
| 10 | 38.3 | 1133.3 | 1133.3 |
| 11 | 47.0 | 415.3 | 415.3 |
| 12 | 68.2 | 2106.4 | 2106.4 |
| 13 | 40.1 | 1524.9 | 1524.9 |
| 14 | 51.4 | 1946.3 | 1946.3 |
| 15 | 53.8 | 3120.1 | 928.9 |
| 16 | 60.7 | 3550.9 | 508.1 |

*Estimates are based on a survey conducted in August 2021 among 2288 individuals from 382 randomly selected households, approximately one year after the first round of vaccination. The sample size was selected to estimate the coverage of at least 1 dose of OCV over the entire town, with a statistical power of 90% and an accuracy of approximately 5% for a coverage of 70%. The survey was not powered to estimate coverage at the health area or cluster level. For clusters 3, 11, 13, and 16, which encompass parts of several health areas, coverage estimates per health area were transformed into coverage estimates per cluster based on geographic area overlaps, assuming a constant population density. Pre-printed manuscript, including link to data repository: Koyuncu et al., 2024:* <https://osf.io/preprints/osf/fgq6e> [1]

**Table B** Annual proportion of patients tested via rapid diagnostic tests among those admitted (% testing positive among those tested).

| **Cluster** | **2017** | | **2018** | | **2019** | | **2020** | | **2021** | |
| --- | --- | --- | --- | --- | --- | --- | --- | --- | --- | --- |
| **1** | 72 | (11) | 25 | (0) | 88 | (26) | 100 | (50) | 86 | (33) |
| **2** | 60 | (52) | 33 | (17) | 65 | (36) | 86 | (41) | 86 | (100) |
| **3** | 68 | (26) | 58 | (36) | 80 | (31) | 100 | (33) | 72 | (16) |
| **4** | 64 | (34) | 65 | (32) | 81 | (27) | 81 | (40) | 90 | (40) |
| **5** | 55 | (63) | 70 | (39) | 63 | (34) | 87 | (47) | 94 | (16) |
| **6** | 83 | (29) | 60 | (32) | 82 | (31) | 96 | (45) | 83 | (60) |
| **7** | 77 | (50) | 57 | (46) | 75 | (39) | 93 | (43) | 88 | (50) |
| **8** | 93 | (20) | 70 | (70) | 75 | (50) | 75 | (33) | 80 | (33) |
| **9** | 63 | (43) | 76 | (27) | 74 | (32) | 92 | (37) | 98 | (18) |
| **10** | 77 | (21) | 72 | (71) | 78 | (40) | 82 | (25) | 92 | (30) |
| **11** | 67 | (31) | 61 | (41) | 69 | (45) | 78 | (30) | 88 | (15) |
| **12** | 61 | (35) | 71 | (64) | 83 | (42) | 68 | (25) | 70 | (42) |
| **13** | 73 | (25) | 68 | (25) | 63 | (40) | 79 | (43) | 95 | (34) |
| **14** | 89 | (36) | 60 | (19) | 62 | (37) | 100 | (27) | 80 | (23) |
| **15** | 85 | (36) | 72 | (32) | 64 | (38) | 68 | (34) | 85 | (46) |
| **16** | 69 | (33) | 58 | (40) | 43 | (48) | 43 | (54) | 95 | (34) |
| **Town** | 71 | (34) | 63 | (38) | 71 | (37) | 82 | (38) | 89 | (33) |

**Table C** Yearly incidence of suspected and confirmed cholera per 1,000 residents.

| **SUSPECTED CHOLERA INCIDENCE PER 1,000** | | | | | | |  |  |  | |  |  |
| --- | --- | --- | --- | --- | --- | --- | --- | --- | --- | --- | --- | --- |
| **Cluster** | **2017** | **2018** | **2019** | **2020** | **2021** | **Min** | **Max** |  |  | |  |  |
| **1** | 7.7 | 1.2 | 3.3 | 1.3 | 1.5 | 1.2 | 7.7 |  |  | |  |  |
| **2** | 13.3 | 6.8 | 5.9 | 4.7 | 2.2 | 2.2 | 13.3 |  |  | |  |  |
| **3** | 7.9 | 4.6 | 4.1 | 3.9 | 1.2 | 1.2 | 7.9 |  | **Colour scale** | |  |  |
| **4** | 13.1 | 8.6 | 5.9 | 4.3 | 0.9 | 0.9 | 13.1 |  |  |  |  |  |
| **5** | 5.0 | 3.4 | 4.1 | 2.4 | 2.3 | 2.3 | 5.0 |  | \| 0.0 \| \| --- \| \| 2.5 \| \| 5.0 \| \| 6.0 \| \| 7.5 \| \| 10.0 \| \| 12.5 \| | |  |  |
| **6** | 10.3 | 6.9 | 5.3 | 2.8 | 1.4 | 1.4 | 10.3 |  |  |  |  |  |
| **7** | 2.2 | 2.1 | 1.1 | 1.9 | 1.1 | 1.1 | 2.2 |  |  |  |  |  |
| **8** | 0.8 | 0.9 | 0.8 | 0.3 | 0.5 | 0.3 | 0.9 |  |  |  |  |  |
| **9** | 10.7 | 13.0 | 12.2 | 10.3 | 2.6 | 2.6 | 13.0 |  |  |  |  |  |
| **10** | 3.0 | 2.7 | 3.6 | 3.5 | 2.0 | 2.0 | 3.6 |  |  |  |  |  |
| **11** | 2.0 | 1.8 | 1.7 | 2.4 | 0.8 | 0.8 | 2.4 |  |  |  |  |  |
| **12** | 3.2 | 3.5 | 2.9 | 3.0 | 0.5 | 0.5 | 3.5 |  |  | |  |  |
| **13** | 2.5 | 2.6 | 1.2 | 2.1 | 1.1 | 1.1 | 2.6 |  |  | |  |  |
| **14** | 3.6 | 4.8 | 2.0 | 3.1 | 1.5 | 1.5 | 4.8 |  |  | |  |  |
| **15** | 7.0 | 4.1 | 3.7 | 4.0 | 1.5 | 1.5 | 7.0 |  |  | |  |  |
| **16** | 5.0 | 6.7 | 7.2 | 10.6 | 5.4 | 5.0 | 10.6 |  |  | |  |  |
| **Min** | 0.8 | 0.9 | 0.8 | 0.3 | 0.5 |  |  |  |  | |  |  |
| **Max** | 13.3 | 13.0 | 12.2 | 10.6 | 5.4 |  |  |  |  | |  |  |
|  |  |  |  |  |  |  |  |  |  | |  |  |
| **CONFIRMED CHOLERA INCIDENCE PER 1,000** | | | | | | |  |  | |  | |  |
| **Cluster** | **2017** | **2018** | **2019** | **2020** | **2021** | **Min** | **Max** |  | |  | |  |
| **1** | 1.2 | 0.0 | 0.9 | 0.7 | 0.4 | 0.0 | 1.2 |  | |  | |  |
| **2** | 4.8 | 0.4 | 1.1 | 1.7 | 1.7 | 0.4 | 4.8 |  | |  | |  |
| **3** | 2.7 | 0.7 | 1.1 | 1.7 | 0.3 | 0.3 | 2.7 |  | |  | |  |
| **4** | 6.0 | 2.5 | 2.0 | 1.7 | 0.4 | 0.4 | 6.0 |  | | **Colour scale** | |  |
| **5** | 1.7 | 1.1 | 1.3 | 1.1 | 1.1 | 1.1 | 1.7 |  | |  |  |  |
| **6** | 4.0 | 2.0 | 1.6 | 1.1 | 0.7 | 0.7 | 4.0 |  | | \| 0.0 \| \| --- \| \| 1.0 \| \| 2.0 \| \| 3.0 \| \| 4.0 \| \| 5.0 \| \| 6.0 \| | |  |
| **7** | 1.0 | 0.5 | 0.5 | 0.8 | 0.5 | 0.5 | 1.0 |  | |  |  |  |
| **8** | 0.2 | 0.4 | 0.3 | 0.1 | 0.2 | 0.1 | 0.4 |  | |  |  |  |
| **9** | 3.9 | 3.0 | 4.2 | 4.4 | 1.3 | 1.3 | 4.4 |  | |  |  |  |
| **10** | 0.5 | 1.1 | 1.3 | 1.2 | 1.3 | 0.5 | 1.3 |  | |  |  |  |
| **11** | 0.5 | 0.6 | 0.6 | 1.0 | 0.3 | 0.3 | 1.0 |  | |  |  |  |
| **12** | 0.8 | 1.2 | 1.1 | 0.9 | 0.1 | 0.1 | 1.2 |  | |  |  |  |
| **13** | 0.6 | 0.5 | 0.4 | 0.9 | 0.5 | 0.4 | 0.9 |  | |  | |  |
| **14** | 1.3 | 0.6 | 0.6 | 1.4 | 0.5 | 0.5 | 1.4 |  | |  | |  |
| **15** | 2.6 | 0.8 | 0.7 | 2.0 | 0.9 | 0.7 | 2.6 |  | |  | |  |
| **16** | 1.7 | 1.2 | 0.8 | 3.9 | 2.6 | 0.8 | 3.9 |  | |  | |  |
| **Min** | 0.2 | 0.0 | 0.3 | 0.1 | 0.1 |  |  |  | |  | |  |
| **Max** | 6.0 | 3.0 | 4.2 | 4.4 | 2.6 |  |  |  | |  | |  |

References:

1. Koyuncu A, Bugeme P, Hulse JD, Hutchins C, Xu H, Gallandat K, et al. Challenges with Achieving and Maintaining High Oral Cholera Vaccine Coverage in Uvira, The Democratic Republic of the Congo: serial cross-sectional representative surveys. OSF Pre-Print (2024, January 9). https://doi.org/10.31219/osf.io/fgq6e
