# Supplementary material for: Improved water supply infrastructure to reduce acute diarrhoeal diseases and cholera in Uvira, Democratic Republic of the Congo: Results and lessons learned from a pragmatic trial
Source: PLoS Negl Trop Dis. 2024 Jul 3;18(7):e0012265. doi: 10.1371/journal.pntd.0012265 (PMC11251581; doi:10.1371/journal.pntd.0012265)

**SUPPLEMENTAL INFORMATION**

**“S1 FIGURES”**

**Fig A** Monthly average number of suspected and confirmed cases from each cluster.


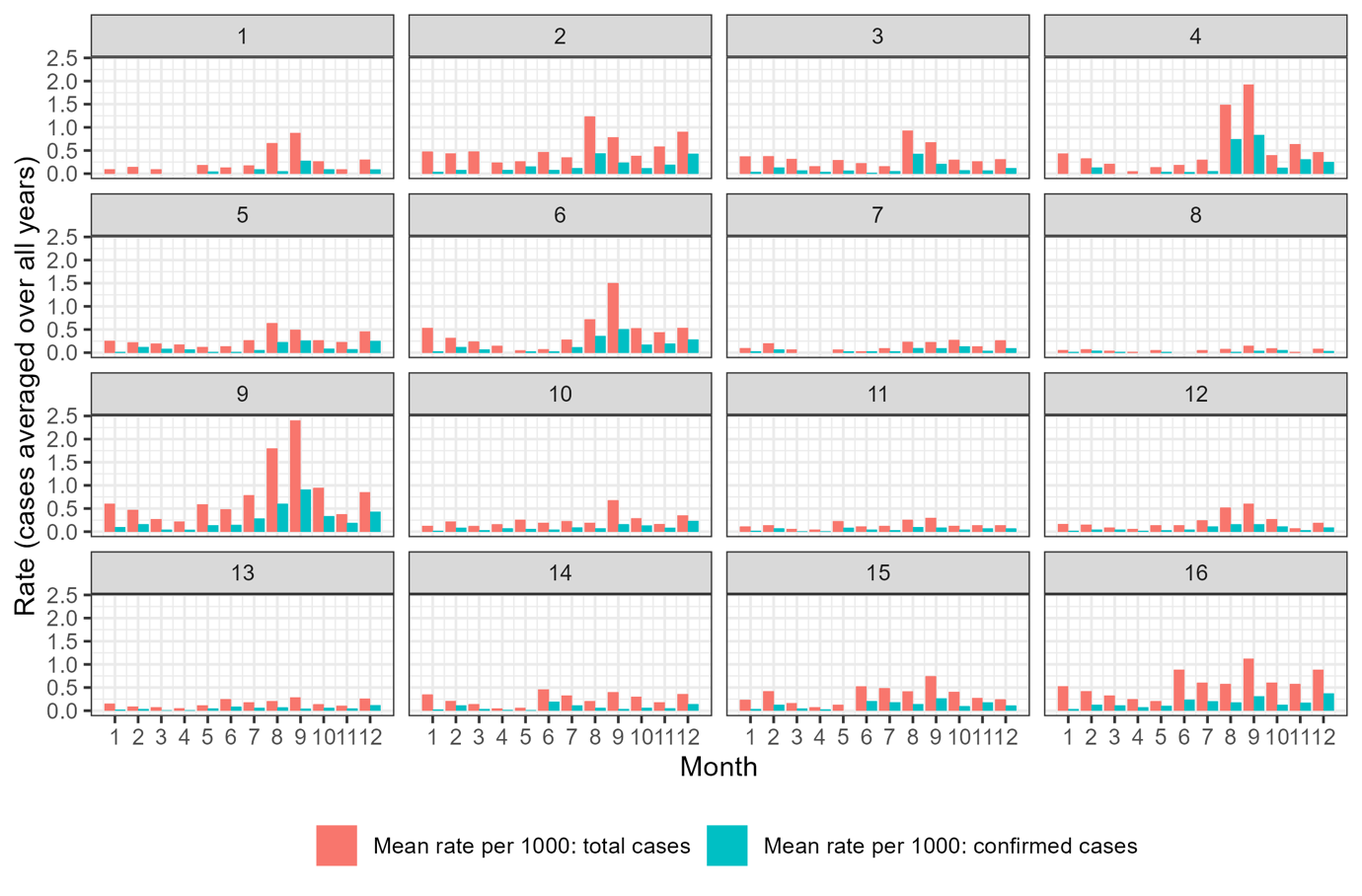


**Fig B** Description of water supply-related exposures: monthly mean accessibility (%), water quantity (L/cap/day), affordability (%), and overall service quality (composite index, %) by cluster and continuity (%) for the town.


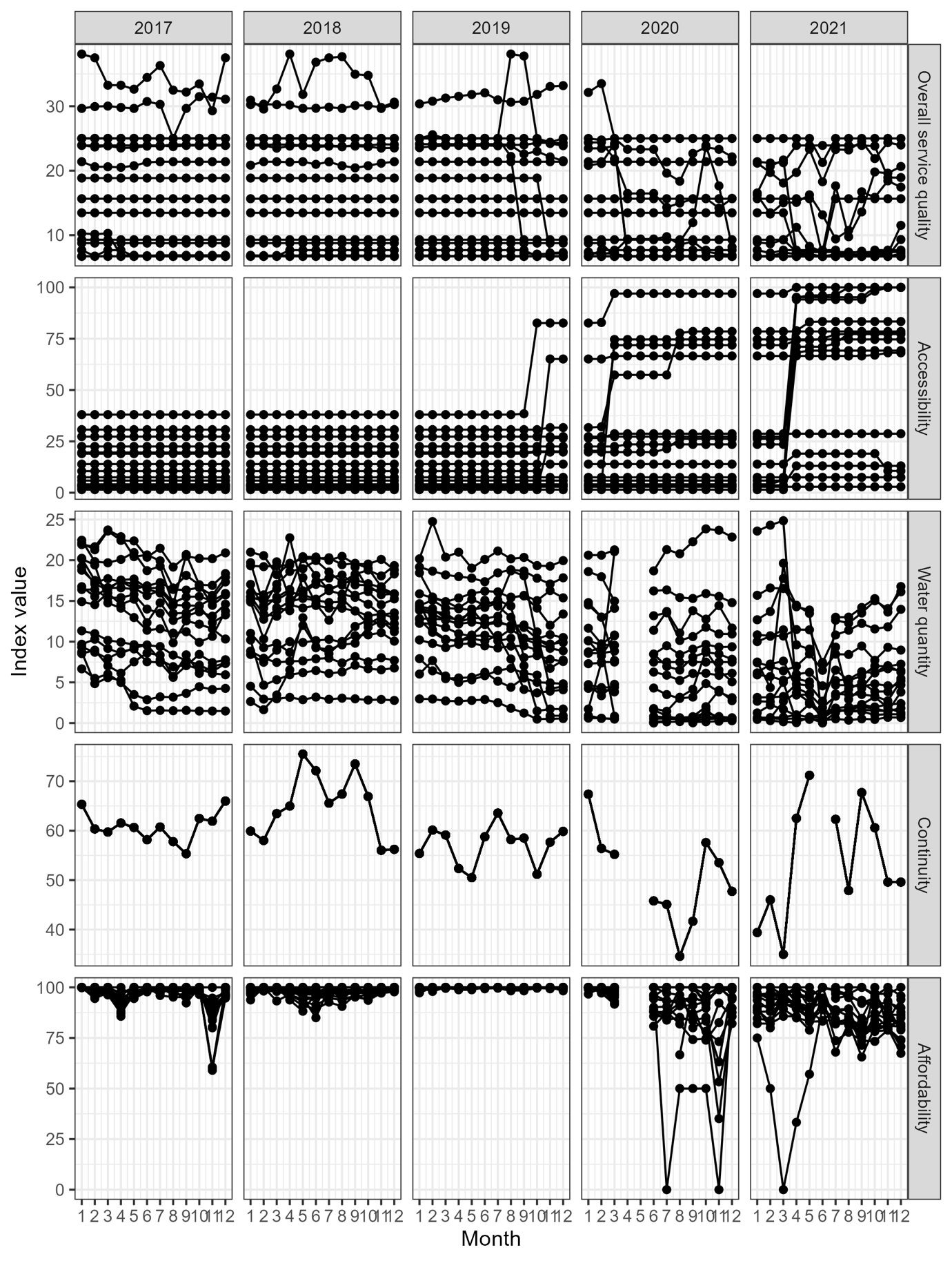


**Fig C** Sensitivity analysis comparing two approaches to modelling time: (i) on the left, “factor” refers to a model including a categorical form for year, which interacts with the harmonic terms for month – this allows for a different seasonal pattern each year and does not assume a linear or quadratic secular trend; (ii) on the right, “quadratic” refers to the model presented in the main manuscript, where harmonic terms for month, a linear and quadratic term for year, and an interaction between the linear term for time and the harmonic terms for year are included.


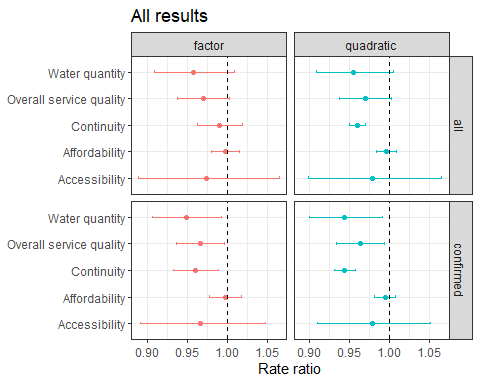

Supplement: S1 Fig — Fig A. Monthly average number of suspected and confirmed cases from each cluster. Fig B: Description of water supply-related exposures: monthly mean accessibility (%), water quantity (L/cap/day), affordability (%), and overall service quality (composite index, %) by cluster and continuity (%) for the town. Fig C: Sensitivity analysis comparing two approaches to modelling time. (DOCX) [file pntd.0012265.s004.docx]
